# Clinical Outcomes of Polyetheretherketone (PEEK) Hybrid Prosthesis in All-on-Four Rehabilitation: A Systematic Review Protocol

**DOI:** 10.12688/f1000research.150799.1

**Published:** 2024-05-17

**Authors:** Hanen Boukhris, Hayet Hajjami, Souha Ben youssef

**Affiliations:** 1 faculty of dental medicine-department of prosthodontics-LR12SP10, University of Monastir, Monastir, Monastir, 4000, Tunisia; 2 Faculty of dental medicine, department of oral surgery, LR12SP10, University of Monastir, Monastir, Monastir, 4000, Tunisia

**Keywords:** All-on-four, PEEK (Polyetheretherketone), Full-arch implant, Systematic review, Dental materials, Success rates, Complications, Clinical outcomes

## Abstract

**Background:**

The “all-on-four” concept represents a significant advancement in dental implantology. particularly beneficial in cases of extensive jaw bone loss where invasive bone regeneration procedures are typically required. However, the successful implementation of this technique necessitates meticulous planning concerning implant selection, materials, and prosthesis design. The recent introduction of PEEK (Polyetheretherketone) in dentistry, especially in all-on-four prosthetics, prompts questions regarding its clinical efficacy and comparative biomechanical and biological advantages over conventional materials such as titanium and zirconia. While some studies have compared PEEK with other materials, systematic reviews evaluating its efficacy are scarce. This systematic review protocol intends to assess the evidence regarding the viability of PEEK as a potential alternative within the all-on-four approach in dental implantology.

**Methods:**

This systematic review protocol will adhere to the Cochrane Handbook for Systematic Review of Interventions and align with the Methodological Expectations of the Cochrane Intervention Reviews (MECIR) guidelines. Utilizing a comprehensive search strategy, multiple databases, including PubMed, EMBASE, Scopus, EBSCO, Web of Science, Cochrane Central, and registries of clinical trials, will be explored. The search aims to identify randomized controlled trials and non-randomized studies investigating the application of PEEK in the all-on-four approach for dental procedures. Emphasizing clinically relevant outcomes such as implant survival, prosthesis success, peri-implant complications, and patient satisfaction, this review aims to provide insights into the effectiveness and potential benefits of using PEEK in all-on-four prosthetics. Non-randomized studies will be assessed for bias using ROBINS-I, while randomized controlled trials will undergo evaluation with the Cochrane Risk of Bias assessment tool, ROB II.

**Discussion:**

The outcomes derived from this systematic review hold great significance for dental practitioners exploring the all-on-four concept. Understanding PEEK’s advantages and limitations compared to titanium and zirconia facilitates tailored treatment plans, enhancing success and satisfaction, ultimately improving dental care quality.

**Systematic review registration:**

PROSPERO: CRD42024531175 (Registered on 13/04/2024).

## Introduction

In the field of clinical dentistry, the merging of three-dimensional (3D) printing methods introduces a pioneering challenge in restorative dentistry. Leading this evolution are techniques such as digital light processing and stereolithography. ^1^^–^^3^ Despite rapid technological advancements propelled by patent expirations and widespread acceptance in dentistry, their adoption in clinical practice hinges primarily on the availability of suitable materials. These materials must ensure precise fabrication and possess requisite biological and physical properties. ^3^ Polymeric materials, notably ultra-high-performance variants like PEKK and PEEK, have garnered attention for their outstanding mechanical properties. PEEK, established in medical applications since the 1990s, has demonstrated success in various procedures, such as spinal fusions and orthopedic surgery. ^4^^–^^6^ The potential utilization of these materials in dental prostheses and implants, including the “all-on-four concept,” has attracted considerable interest from both researchers and clinicians. ^7^^,^^8^

The “all-on-four concept” offers a simplified approach to rehabilitation, introduced in the early 2000s to provide a fixed, comfortable solution for fully edentulous patients with atrophic jaws, while avoiding costly and complex regenerative procedures. ^9^^,^^10^

This method has evolved over time, initially utilizing fused metallic frameworks or milled bars for prostheses construction. Traditionally, titanium or cast metal alloy frameworks were favored for their mechanical properties. However, despite initial success rates, issues such as prosthesis fractures, particularly with polymethylmethacrylate (PMMA) prostheses designed for maxillary use, have prompted exploration of alternative materials. ^11^^–^^13^ Recent suggestions to incorporate polymers into all-on-four prostheses aim to enhance patient satisfaction and confidence, addressing challenges associated with prosthesis fractures and failures. ^14^^,^^15^

Therefore, this proposed systematic review seeks to gather data on the clinical performance of PEEK as a restorative material for all-on-four prostheses, in comparison to alternative materials such as titanium and zirconia. This survey is crucial for dentists. It provides essential data for making informed decisions regarding the utilization of PEEK in the all-on-four concept.

## Protocol

### Methods/design

The systematic review will follow the Preferred Reporting Items for Systematic Review and Meta-analysis Protocols (PRISMA-P) checklist. ^16^^–^^18^ It will also adhere to guidelines outlined in the Methodological Expectations of Cochrane Intervention Reviews and Cochrane Handbook for Systematic Review of Interventions. ^19^^,^^20^ The protocol has been officially recorded in the International Prospective Register of Systematic Reviews (PROSPERO) under registration number CRD42024531175 on 13/04/2024.

### Objectives

**Primary objectives:**

• To evaluate the efficacy of PEEK (Polyetheretherketone) in the all-on-four concept, comparing it to conventional materials like titanium and zirconia.
• To examine related clinical outcomes, including implant survival rates, prosthesis success, patient satisfaction levels, and incidences of peri-implant complications.

**Secondary objectives:**

• Identify any potential advantages or limitations of using PEEK in all-on-four prosthetics compared to traditional materials.
• Provide evidence-based recommendations for clinicians regarding the selection of materials for all-on-four procedures.
• Identify any gaps in the existing literature and suggest potential directions for future research.

### Eligibility criteria

The eligibility criteria are organized based on the PICOS framework (Participant-Intervention-Comparison-Outcome-Study Design). This framework aims to identify the key components of the research question.

The research question for this systematic review is: Does the choice of prosthetic material, especially PEEK (Polyetheretherketone), impact success rates in all-on-four dental implant concept compared to titanium and zirconia?

✓ **Types of participants:** This systematic review will focus on patients who have received all-on-four implant-supported fixed prostheses. Eligible participants must not have medical conditions that could affect bone healing or metabolism, such as diabetes, hypertension, or cardiovascular diseases. However, smokers or those who have undergone chemotherapy or radiation therapy will be excluded due to potential impacts on bone healing.
✓ **Types of interventions:** This systematic review will center on the use of PEEK (Polyetheretherketone) as the prosthetic material in all-on-four concepts. The minimum follow-up period will be six months. The analysis will focus on evaluating the effectiveness of PEEK relative to conventional materials.
✓ **Types of outcomes:** The most important outcome will be the success rate of all-on-four prosthesis. Success will be assessed based on criteria including implant stability, peri-implant soft tissue health, prosthetic stability and function, patient satisfaction, and absence of major complications such as peri-implantitis. These assessments will be conducted at various time points.
**Measures of effect:** The main outcome (success rate) will be quantified using the risk ratio (relative risk) or odds ratio, depending on the available data in the included studies. These measures will evaluate the likelihood of success with PEEK prosthetic material compared to alternative materials in all-on-four rehabilitations.
  Additionally, risk differences and the number needed to treat may be calculated to provide further insights into the clinical significance of any differences observed in success rates between the two groups.
✓ **Study types:** This systematic review will involve randomized controlled trials as well as non-randomized controlled trials (non-RCTs). Additionally, observational studies, including cohort and case-control studies, will be considered if they adhere to at least 15 criteria from the STROBE quality guide. ^21^^–^^23^ Furthermore, comparative studies, such as quasi-experimental designs, will be included if they compare outcomes of all-on-four rehabilitation using PEEK prosthetic material with alternative materials. Exclusion criteria will involve individual case reports and letters to the editor due to limited generalizability, while non-English studies will be excluded to mitigate language bias and resource constraints. Additionally, animal studies will not be considered. Studies with insufficient outcome data will also be excluded to ensure the reliability of the review.

### Search strategy

The improved search strategy will utilize both keywords and controlled vocabulary terms specific to the topic of interest, ensuring a thorough exploration of relevant literature.

In addition to MEDLINE, other databases such as EMBASE, Scopus, EBSCO, Cochrane Central and Web of Science will be systematically searched to minimize the risk of missing relevant studies.

To ensure inclusivity, efforts will be made to identify unpublished literature and ongoing clinical trials through sources such as conference proceedings, dissertations, and clinical trial registries. The advisory board will play a crucial role in guiding the identification of grey literature sources and analyzing their relevance to the review.

Moreover, the search strategy will be complemented by manually searching through the bibliographies of included studies to identify additional articles that may not have been identified through electronic searches alone. This approach will help to minimize the risk of publication bias and ensure an exhaustive overview of the existing evidence.

### Study selection (screening process)

After conducting a thorough search across all identified databases and removing duplicate papers, two reviewers will independently assess the titles and abstracts of potentially eligible articles. Articles meeting the initial eligibility criteria will undergo a full-text examination. Additionally, reviewers will carefully examine the bibliography of selected articles to identify further relevant studies. Papers meeting the selection criteria will proceed to the data extraction stage.

Any differences between the reviewers during the selection phase will be addressed through discussion. If needed, an additional reviewer (SB) will be engaged to assist in reaching a consensus and maintaining the integrity of the selection process.

The findings of the search and selection process will be meticulously documented and reported in the final systematic review, ensuring compliance with the Preferred Reporting Items for Systematic Reviews and Meta-analyses (PRISMA) guidelines. ^19^^,^^20^

### Assessment of methodological quality and risk of bias

To ensure a thorough evaluation of bias within the included studies, two review authors (HB, HH) will independently conduct assessments using the revised Cochrane Risk of Bias Assessment Tool (ROBII) and the Newcastle-Ottawa Scale (NOS). ^24^^,^^25^ These established tools will facilitate our analysis of multiple aspects, including participant recruitment, classification of interventions, adherence to intended protocols, data completeness, outcome assessment, and reporting of results.

Each identified risk will be categorized as low, high, serious, or moderate, allowing for a nuanced understanding of the quality of evidence.

If there are any discrepancies between the review authors, they will be addressed through thorough comprehensive discussion or, if required, consultation with a third reviewer.

### Data extraction

Two independent reviewers (HB, HH) will conduct data extraction using a template designed for this purpose, which has been carefully evaluated during a pilot phase.

In the data extraction phase, any discrepancies will be addressed through discussion by the two reviewers as the initial step. If consensus cannot be achieved, (SB) will be consulted for further input. Final decisions will be made based on majority agreement. In instances where information or data is unpublished or missing, attempts will be made to contact the study authors to request the necessary details. If sufficient data are obtained, additional pertinent statistical analyses will be conducted.

### Data analysis and synthesis

The analysis of results and data will involve descriptive statistics, such as mean and median, along with measures of dispersion. Frequencies and proportions will be calculated for all variables. The findings will be presented through both textual descriptions and tables, utilizing narrative synthesis.

Subgroup analyses may be conducted if feasible, and sensitivity analyses will assess the robustness of findings.

If considered suitable, a meta-analysis will be conducted utilizing random-effects models to combine data from studies with methodology and outcome homogeneity. To assess statistical heterogeneity, comprehensive tests like the Chi-squared, Tau-squared, and I-squared metrics will be used. Heterogeneity levels will be systematically categorized, from low to high or severe, to interpret observed variability. These findings will be illustrated through a forest plot, providing a clear visualization of combined study effects. In identifying potential sources of heterogeneity, thorough subgroup analyses will be conducted. These analyses will examine key characteristics impacting incidence or prevalence estimates. The aim is to enhance the objectivity of the findings by investigating these factors thoroughly.

Moreover, to address potential publication bias, a funnel plot will be utilized, grouping studies in sets of ten for meta-analysis. The plot’s symmetry will then be carefully assessed using the Egger test to identify any bias in the included studies.

## Discussion

The findings of this proposed systematic review hold importance for dentists specializing in dental implantology. They will play a critical role in enhancing patient care for the all-on-four concept.

By consolidating existing evidence on PEEK’s use in all-on-four prosthetics, this review helps clinicians make informed decisions about material selection. Understanding both the potential benefits and limitations of PEEK compared to traditional materials like titanium and zirconia allows dentists to personalize treatment plans, enhancing success and satisfaction.

Consequently, this review serves as a valuable resource for guiding clinical practice and elevating the quality of dental care provided.

### Ethics and dissemination

No ethical approval is needed for this systematic survey. The authors intend to present the findings at target conferences and publish the research findings in a peer-reviewed journal adopting open science practices.

### Study status

This systematic review is currently in the data analysis process. The protocol of this systematic review was submitted to PROSPERO registry on 13th April, 2024 (CRD42024531175).

## Data Availability

No data are associated with this article. Figshare: PRISMA-P checklist for Clinical Outcomes of Polyetheretherketone (PEEK) Hybrid Prosthesis in All-on-Four Rehabilitation: A Systematic Review https://doi.org/10.6084/m9.figshare.25662519.v1. ^26^ Data are available under the terms of the Creative Commons Attribution 4.0 International license (CC-BY 4.0).
