# Supplementary material for: Clinical Outcomes of Polyetheretherketone (PEEK) Hybrid Prosthesis in All-on-Four Rehabilitation: A Systematic Review Protocol
Source: F1000Res. 2025 Jan 30;13:507. Originally published 2024 May 17. [Version 2] doi: 10.12688/f1000research.150799.2 (PMC13560014; doi:10.12688/f1000research.150799.2)
Supplement: Peer review history

# Reviewer report: Reviewer response for version 2

**DOI:** 10.5256/f1000research.177496.r423867

**Published:** 2026-08-18

**Authors:** Omid Panahi

**Affiliations:** 1 Centro Escolar University, Manila, Metro Manila, Philippines

This manuscript presents a systematic review protocol investigating the clinical outcomes of polyetheretherketone (PEEK) hybrid prostheses used in All-on-Four implant rehabilitation. The rationale is clinically relevant because PEEK has increasingly been proposed as an alternative prosthetic framework material, while comparative clinical evidence against conventional materials such as titanium and zirconia remains limited. The authors aim to evaluate implant survival, prosthetic success, peri-implant complications, patient satisfaction, and aesthetic outcomes, while also identifying potential advantages, limitations, and gaps in the existing evidence. The protocol is registered in PROSPERO (CRD42024531175) and states that PRISMA-P and Cochrane methodological guidance will be followed.

 Overall, the research question is relevant, and the proposed systematic-review design is appropriate. The revised version has addressed several concerns raised during the previous peer-review process, particularly the distinction between success and survival, the exclusion of in vitro and animal studies, clarification of outcomes, and expansion of the search strategy. Nevertheless, some methodological aspects should be further clarified before the protocol can be considered fully reproducible and methodologically robust.

 1. Is the rationale for, and objectives of, the study clearly described?

**Yes.**

 The rationale is adequately presented. The introduction explains the clinical relevance of the All-on-Four concept and the increasing interest in PEEK as a prosthetic material. The authors appropriately identify the lack of systematic evidence evaluating the clinical efficacy of PEEK compared with conventional materials.

 The objectives are also clearly stated. The primary objectives include evaluation of PEEK efficacy compared with titanium, zirconia, and other materials, as well as assessment of implant survival, prosthesis success, patient satisfaction, peri-implant complications, and aesthetic outcomes. Secondary objectives address potential advantages and limitations, clinical recommendations, and evidence gaps.

 The research question is also explicitly formulated according to the PICOS framework.

 One minor recommendation is that the authors should ensure complete consistency between the terminology used in the title, research question, objectives, eligibility criteria, and outcomes. In particular, "success" and "survival" should remain clearly separated throughout the protocol, as the authors have already attempted to do in Version 2.

 2. Is the study design appropriate for the research question?

**Yes.**

 A systematic review is an appropriate design for addressing the proposed comparative clinical question because the objective is to synthesize existing evidence regarding PEEK versus alternative prosthetic materials in All-on-Four rehabilitation.

 The protocol appropriately plans to include randomized and non-randomized comparative studies and observational evidence where relevant. It also describes independent study selection, duplicate assessment, risk-of-bias evaluation, data extraction, narrative synthesis, and a possible meta-analysis when sufficient methodological and clinical homogeneity exists.

 However, the authors should strengthen the protocol by prespecifying how different study designs will be handled analytically. In particular, randomized trials and observational studies should not automatically be pooled together without an explicit methodological justification. Separate analyses or clearly defined hierarchical approaches may be necessary depending on the available evidence.

 3. Are sufficient details of the methods provided to allow replication by others?

**Partly.**

 The protocol contains many important methodological elements, including the databases to be searched, eligibility criteria, study-selection procedures, duplicate data extraction, approaches to resolving disagreements, risk-of-bias assessment, and proposed statistical synthesis.

 However, some important details remain insufficiently specified for complete independent replication.

 Major methodological points that should be addressed

**1. Exact search strategies should be provided.**

 The manuscript states that keywords and controlled vocabulary will be used and that the search strategy will be clearly defined, but the actual database-specific search strings are not presented in the protocol text.

 The authors should provide the complete search strategy for at least MEDLINE/PubMed and preferably all major databases, including Boolean operators, MeSH/controlled vocabulary, truncation, limits, and the date on which each database will be searched. This is essential for reproducibility.

**2. Risk-of-bias tools need clarification and consistency.**

 The abstract states that non-randomized studies will be assessed using ROBINS-I and randomized trials using RoB 2, whereas the Methods section states that the authors will use RoB 2 and the Newcastle-Ottawa Scale (NOS).

 This discrepancy should be resolved. The authors should specify exactly which risk-of-bias instrument will be used for each eligible study design and provide a methodological justification for that choice.

**3. Eligibility criteria require further clarification.**

 The protocol excludes patients with conditions such as diabetes, hypertension, and cardiovascular disease, while also excluding smokers and patients receiving chemotherapy or radiotherapy.

 The rationale for these exclusions should be explicitly justified and supported by appropriate evidence. In particular, the authors should clarify whether these characteristics are exclusion criteria for individual studies or whether studies are excluded only when the entire study population meets these criteria. This distinction could substantially affect study selection and external validity.

**4. Outcome definitions should be prespecified in greater detail.**

 Although the manuscript distinguishes survival from success, the precise operational definitions and measurement time points should be specified for each primary and secondary outcome.

 For example, the authors should specify how prosthetic success, implant survival, peri-implant complications, prosthetic complications, and aesthetic outcomes will be defined and what instruments or validated criteria will be accepted.

**5. The statistical analysis plan needs greater specificity.**

 The manuscript proposes descriptive statistics, narrative synthesis, subgroup and sensitivity analyses, and a random-effects meta-analysis when appropriate. Heterogeneity will be assessed using Chi-squared, Tau-squared, and I-squared statistics.

 The authors should prespecify:

- the minimum degree of methodological/clinical comparability required for pooling;
- the effect measures for each outcome;
- how continuous outcomes will be handled;
- how studies with different follow-up periods will be analyzed;
- how missing data will be treated;
- how zero-event studies will be handled;
- criteria for conducting subgroup and sensitivity analyses; and
- how different study designs will be incorporated into quantitative synthesis.

**6. The approach to publication bias should be revised or clarified.**

 The manuscript proposes funnel plots and Egger's test and states that studies will be grouped in sets of ten.

 The rationale for this "sets of ten" approach is unclear. The authors should clarify the minimum number of studies required before funnel-plot asymmetry and Egger's test are performed and explain the methodological basis for this decision.

**7. Data extraction should be described more comprehensively.**

 The manuscript states that two reviewers will independently extract data using a pilot-tested template.

 For reproducibility, the authors should specify the principal variables to be extracted, including participant characteristics, jaw location, number and distribution of implants, prosthetic design, PEEK formulation/manufacturing technique, comparator material, loading protocol, follow-up duration, outcome definitions, complications, and funding/conflict-of-interest information.

 4. Are the datasets clearly presented in a useable and accessible format?

**Not applicable.**

 This is a systematic review protocol rather than a completed systematic review with an underlying clinical dataset. The manuscript explicitly states that no data are associated with the article.

 Therefore, assessment of dataset accessibility is not applicable at the protocol stage. Nevertheless, when the completed systematic review is indexed, the authors should consider making the extracted dataset, analysis code, and supplementary search strategies available where possible to maximize transparency and reproducibility.

**Overall assessment**

 This is a clinically relevant and potentially useful systematic review protocol addressing an important question in implant prosthodontics and dental materials. The rationale, research question, and objectives are adequately described, and the systematic-review design is appropriate.

 The manuscript has made meaningful improvements compared with the earlier version, particularly regarding the distinction between success and survival, clinical eligibility criteria, search strategy, and outcome assessment.

 However, **the methodological reporting should be strengthened before the protocol can be considered fully reproducible**. The most important issues that should be addressed are the inconsistency between ROBINS-I and NOS, provision of complete reproducible search strategies, more precise operational definitions of outcomes, clarification of eligibility criteria, pre specification of the statistical synthesis plan, and clarification of the approach to publication bias.

 Points that must be addressed for scientific soundness

1. **Resolve the inconsistency in risk-of-bias tools (ROBINS-I versus NOS) and specify the tool for each study design.**
2. **Provide complete, reproducible database-specific search strategies.**
3. **Clarify and justify the eligibility/exclusion criteria, particularly medical comorbidities and smoking.**
4. **Provide operational definitions and measurement methods for all primary and secondary outcomes.**
5. **Prespecify the criteria and statistical methods for meta-analysis, subgroup analysis, sensitivity analysis, and handling of different study designs.**
6. **Clarify the methodology and minimum-study threshold for funnel plots and Egger's test.**

Addressing these points would substantially improve methodological transparency, reproducibility, and confidence in the validity of the eventual systematic-review findings.

**Overall recommendation: The protocol is potentially suitable for indexation, but the above methodological clarifications should be addressed to ensure full scientific rigor and reproducibility.**

Is the study design appropriate for the research question?

Yes

Is the rationale for, and objectives of, the study clearly described?

Yes

Are sufficient details of the methods provided to allow replication by others?

Partly

Are the datasets clearly presented in a useable and accessible format?

Not applicable

Reviewer Expertise:

Fixed Prosthodontics; Dental Materials; Implant Dentistry; Digital Dentistry; Dental Implant Biomechanics; Systematic Reviews and Evidence-Based Dentistry.

I confirm that I have read this submission and believe that I have an appropriate level of expertise to confirm that it is of an acceptable scientific standard, however I have significant reservations, as outlined above.

# Reviewer report: Reviewer response for version 1

**DOI:** 10.5256/f1000research.165398.r350243

**Published:** 2025-01-17

**Authors:** Marwa Emam

**Affiliations:** 1 Fixed Prosthodontics, Ain Shams University, Cairo, Egypt

The report contains minor issues regarding the clarity of its English language. Editing is required to improve the flow and enhance the understandability of the sentences.

**Introduction Section:**

- It is unclear whether only *in vivo* studies will be included, as outcomes cannot be effectively investigated through *in vitro* studies.
- The first sentences of the introduction should focus on implant dentistry or the available dental materials for CAD/CAM manufacturing, rather than 3D printing manufacturing.
- There is no need to mention PEKK since it will not be included in the review. Additionally, abbreviations should only be used after the full term has been introduced.
- A brief mention of the material’s composition and its related properties should be included here.
- PMMA is primarily a provisional material, so comparing it with PEEK is somewhat inappropriate. Instead, titanium and Co-Cr should be highlighted as they are considered the gold standard. Mention the advantages zirconia and PEEK bring to clinical outcomes.

**Objectives (Primary Objectives):**

- Have you considered including base metals?
- How will each outcome be measured, and will aesthetic success be evaluated?

**Eligibility Criteria:**

- There is a significant difference between “success” and “survival.” The report should specify the measured outcomes, and the terms should not be used interchangeably.

**Measures of Effect:**

- The methods used to assess all parameters should be clearly defined and explicitly stated.

Is the study design appropriate for the research question?

Partly

Is the rationale for, and objectives of, the study clearly described?

Partly

Are sufficient details of the methods provided to allow replication by others?

Yes

Are the datasets clearly presented in a useable and accessible format?

Yes

Reviewer Expertise:

Fixed prosthodontics, dental materials, digital dentistry, implant biomechanics.

I confirm that I have read this submission and believe that I have an appropriate level of expertise to confirm that it is of an acceptable scientific standard, however I have significant reservations, as outlined above.

# Response

**Published:** 2025-01-18

**Authors:** Hanen Boukhris

**Affiliations:** university hospital F- Hached, faculty of dental medicine university of monastir tunisia, sousse, tunisia, Tunisia

Dear Reviewer,

 Thank you for your valuable feedback on our systematic review protocol. We appreciate your careful consideration of the manuscript and your insightful comments, which will help improve the clarity and precision of the report. Below, we address each of your reservations:

1. **Clarity of English Language:**
  - We recognize the importance of clear and concise language in scientific writing. We will thoroughly revise the manuscript to improve the flow and readability, ensuring that sentences are more straightforward and easier to follow. A native English speaker will assist with the final editing to enhance the language quality further.
2. **Introduction Section:**
  - **In vivo vs. In vitro studies:** We understand the need for clarification regarding the inclusion criteria for in vivo studies. We will revise the introduction to explicitly state that only in vivo studies will be included in the review, as outcomes cannot be effectively evaluated through in vitro studies.
  - **Focus on Implant Dentistry:** We acknowledge that the introduction should primarily focus on implant dentistry rather than 3D printing manufacturing. The first sentences will be rephrased to center on available dental materials for CAD/CAM manufacturing, as this aligns better with the focus of the review.
  - **PEKK Mention:** We will remove the mention of PEKK from the introduction as it will not be part of the review, streamlining the discussion.
  - **Material Composition and Properties:** We agree with your suggestion to include a brief mention of PEEK's composition and its relevant properties in the introduction. This information will be incorporated to provide a better context for readers.
3. **Comparison of PMMA with PEEK:**
  - We acknowledge that PMMA is primarily a provisional material, and comparing it with PEEK may not be entirely appropriate. In light of this, we will revise the text to focus more on titanium and Co-Cr alloys as the gold standard materials for all-on-four prosthetics. Additionally, we will highlight the clinical advantages that zirconia and PEEK bring to the outcomes of such treatments.
4. **Objectives (Primary Objectives):**
  - **Inclusion of Base Metals:** We will consider incorporating base metals into the review, as their inclusion may provide a more comprehensive comparison. This will be reflected in the revised primary objectives.
  - **Outcome Measures and Aesthetic Success:** We will provide clearer details on how each outcome will be measured, including the evaluation of aesthetic success, which is crucial for patient satisfaction and clinical outcomes.
5. **Eligibility Criteria:**
  - **Clarification of "Success" vs. "Survival":** We will ensure that the terms "success" and "survival" are clearly defined and not used interchangeably. The manuscript will specify how each outcome is measured, distinguishing between these two concepts.
6. **Measures of Effect:**
  - We will revise the manuscript to clearly define the methods used to assess each outcome, including the rationale behind the selection of these measures. The objectives of the study will be more explicitly stated to ensure clarity.
7. **Study Design Appropriateness:**
  - We will revisit the study design section and provide a more thorough explanation of why the chosen design is appropriate for addressing the research question, ensuring that all methodological details align with the objectives of the review.

We believe that these revisions will strengthen the manuscript and enhance its clarity and precision. Once again, we appreciate your constructive feedback, and we are confident that the revised version will meet your expectations.

 Sincerely,

# Reviewer report: Reviewer response for version 1

**DOI:** 10.5256/f1000research.165398.r300937

**Published:** 2024-10-04

**Authors:** Saja Muhsin

**Affiliations:** 1 Middle Technical University, Baghdad, Baghdad Governorate, Iraq

Summary; Two independent reviewers will use the Cochrane Risk of Bias Tool and the Newcastle-Ottawa Scale to assess studies, addressing discrepancies through discussion or a third reviewer to ensure a comprehensive evaluation of bias. Data extraction will be performed independently, with differences resolved similarly. Descriptive statistics and narrative synthesis will summarize findings, with subgroup and sensitivity analyses enhancing robustness. If feasible, a random-effects meta-analysis will be conducted, using Chi-squared, Tau-squared, and I-squared tests to assess heterogeneity. Funnel plots and the Egger test will evaluate publication bias, aiming for objective and reliable results.

 To ensure the article is scientifically sound, the authors should focus on the following specific points:

 1. Bias Assessment Tools: Justify the choice of the Cochrane Risk of Bias Tool (ROBII) and the Newcastle-Ottawa Scale (NOS) for assessing bias. Explain how these tools complement each other and their relevance to the study design.

 2. Discrepancy Resolution: Provide detailed procedures for resolving discrepancies between reviewers. Specify criteria for involving a third reviewer and how to achieve consensus to enhance transparency.

 3. Data Extraction Template: Include a comprehensive description of the data extraction template and the pilot phase evaluation. This ensures the template's effectiveness and reliability in capturing relevant data.

 4. Unpublished/Missing Data: Outline the specific steps and methods for contacting study authors to obtain unpublished or missing data. Clarify how these data will be integrated into the analysis and the impact on overall findings.

 5. Statistical Analyses: Elaborate on the statistical methods, including how descriptive statistics, frequencies, and proportions will be calculated. Provide clear definitions and justifications for the measures of dispersion used.

 6. Subgroup and Sensitivity Analyses: Detail the criteria for conducting subgroup analyses and the factors considered in sensitivity analyses. Explain how these analyses will contribute to understanding the robustness and generalizability of the findings.

 7. Meta-Analysis Criteria: Specify the criteria for including studies in the meta-analysis, particularly the definitions of methodological and outcome homogeneity. Justify the use of random-effects models and provide a rationale for the selected tests (Chi-squared, Tau-squared, and I-squared) to assess heterogeneity.

 8. Heterogeneity Categorization: Clearly define the categories of heterogeneity (low, high, severe) and explain the thresholds or criteria used to classify them. This aids in interpreting the variability among included studies.

 9. Publication Bias: Provide a detailed methodology for creating and interpreting the funnel plot and using the Egger test. Explain the rationale for grouping studies in sets of ten and how symmetry will be assessed to identify publication bias.

 10. Visualization of Results: Ensure that the forest plot and any other visualizations are clearly described and justified. Explain how these visual tools will aid in the interpretation and presentation of the combined study effects.

 By addressing these points, the authors can strengthen the methodological rigour and scientific validity of their study, making it more robust and reliable.

Is the study design appropriate for the research question?

Yes

Is the rationale for, and objectives of, the study clearly described?

Yes

Are sufficient details of the methods provided to allow replication by others?

Yes

Are the datasets clearly presented in a useable and accessible format?

Yes

Reviewer Expertise:

Dental Material Sciences, Prosthodontics, Research Methodoldy (Dental field)

I confirm that I have read this submission and believe that I have an appropriate level of expertise to confirm that it is of an acceptable scientific standard.

## References

[1] : Heterogeneity in meta-analysis: a comprehensive overview. *Int J Biostat* .2024;20(1) : 10.1515/ijb-2022-0070169-199 10.1515/ijb-2022-0070 36961993
